# Quantitative analysis of diet structure by real-time PCR, reveals different feeding patterns by two dominant grasshopper species

**DOI:** 10.1038/srep32166

**Published:** 2016-08-26

**Authors:** Xunbing Huang, Huihui Wu, Mark Richard McNeill, Xinghu Qin, Jingchuan Ma, Xiongbing Tu, Guangchun Cao, Guangjun Wang, Xiangqun Nong, Zehua Zhang

**Affiliations:** 1 State Key Laboratory of Biology of Plant Diseases and Insect Pests, Institute of Plant Protection, Chinese Academy of Agricultural Sciences, Beijing, 100193, P.R. China; 2 Scientific Observation and Experimental Station of Pests in Xilin Gol Rangeland, Institute of Plant Protection, Chinese Academy of Agricultural Sciences, Xilinhot, 02600, P.R. China; 3 AgResearch, Lincoln Research Centre, Christchurch, 8140, New Zealand

## Abstract

Studies on grasshopper diets have historically employed a range of methodologies, each with certain advantages and disadvantages. For example, some methodologies are qualitative instead of quantitative. Others require long experimental periods or examine population-level effects, only. In this study, we used real-time PCR to examine diets of individual grasshoppers. The method has the advantage of being both fast and quantitative. Using two grasshopper species, *Oedaleus asiaticus* and *Dasyhippus barbipes*, we designed ITS primer sequences for their three main host plants, *Stipa krylovii, Leymus chinensis* and *Cleistogenes squarrosa* and used real-time PCR method to test diet structure both qualitatively and quantitatively. The lowest detection efficiency of the three grass species was ~80% with a strong correlation between actual and PCR-measured food intake. We found that *Oedaleus asiaticus* maintained an unchanged diet structure across grasslands with different grass communities. By comparison, *Dasyhippus barbipes* changed its diet structure. These results revealed why *O. asiaticus* distribution is mainly confined to *Stipa*-dominated grassland, and *D. barbipes* is more widely distributed across Inner Mongolia. Overall, real-time PCR was shown to be a useful tool for investigating grasshopper diets, which in turn offers some insight into grasshopper distributions and improved pest management.

Plant species composition is critically important for herbivores. For example, changes in plant community composition can strongly affect grasshopper biology, including nutrition, development, growth, fecundity^1^,^2^, migration, distribution, population dynamics^3^,^4^,^5^ and even anti-predator defense^6^. Grasshopper diets can be determined both by plant availability and feeding preferences^7^, with host plants divided into preferred, less-preferred, not preferred but sometimes eaten, and rejected plant species and tissues^8^. For example, grasshoppers in the subfamilies Acridinae and Oedipodinae generally prefer monocots and dicotyledons, respectively, whereas the Catantopinae will feed on both^9^.

Generally, grasshopper feeding habits can be determined and broadly categorized by employing field cages^10^,^11^,^12^, examining the morphological structure of grasshopper mouthparts^13^,^14^, analyzing the morphology of grasshopper droppings^11^,^15^,^16^,^17^, or examining the structure of the grasshopper alimentary canal^18^,^19^. Microscopic examination and DNA barcoding of gut content or faeces have also been used to determine the food preference of grasshoppers^20^,^21^,^22^,^23^,^24^,^25^. But these methods have certain limitations, such as long experimental period, lack of quantitative precision, and inability to accurately measure feeding in individuals, where food intake is obviously in comparatively small amounts. Because these approaches may in some cases have little practical application to understanding grasshopper biology, techniques that are more simple and quantitative offer new ways to understand insect biology at an individual level while providing insights into the drivers of population level effects in plant communities.

Of the various techniques, real-time PCR is a highly sensitive technology, with high signal-to-noise ratio and high throughput, which can be used to evaluate insect diets^26^. This technique builds upon conventional PCR by including a fluorescent dye that binds to double-stranded DNA, and thus the quantity of DNA produced in each PCR cycle is monitored using a spectrophotometer during the PCR process^27^. It requires a special thermocycler and specific reagents used for fluorescence, but does not require equipment associated with gel analysis used in conventional PCR, that could detect a higher frequency of insect food consumption than conventional PCR when both tests are run at optimal conditions^28^,^29^. It has been used in pathology and vector insect research^30^,^31^,^32^, to investigate the relationship between humans and mosquitoes^33^, as well as insect classification, insect nucleotide polymorphism, insect toxicology^34^, insect physiology^35^, and mostly focusing on insect molecular ecology^28^,^29^,^36^,^37^,^38^,^39^,^40^ *et al*.

To date, this method has not yet been utilized to study the diet structure of grasshopper. Therefore, to investigate the suitability of this technique vis-à-vis grasshopper feeding, we undertook the following project. We examined food intake and preference of two grasshoppers, *Oedaleus asiaticus* B. Bienko and *Dasyhippus barbipes* (Fischer-Waldheim). These grasshopper species are dominant and devastating pests in Inner Mongolia, China. *O. asiaticus* distribution is mainly confined to *Stipa*-dominated grassland, and *D. barbipes* is more widely distributed across Inner Mongolia. They mainly feed on grasses, particularly *C. squarrosa*, *L. chinensis and S. krylovii*^2^,^41^,^42^. These three gramineous plants (all Poales: Poaceae) comprise > 80% of all above-ground plant biomass at our study site, and are important components of the Xinli Gol Grassland ecosystem, serving to provide habitats for insects and many other animals^43^.

The aim of this study was to determine if real-time PCR could be used to determine grasshopper feeding patterns (diet structure) and food preferences in grasslands with different plant communities. Understanding grasshopper feeding patterns and food preference in different plant communities by this new method is not only useful for determining basic insect nutritional ecology and general ecology, but is important for understanding species distribution across grasslands, which in turn aids the selection of appropriate control management techniques for specific grasshopper species in fragile grassland ecosystems.

## Results

### Plant and grasshopper composition in *Stipa*-dominated grassland and *Leymus*-dominated grassland at our study site

**Figure 1:**
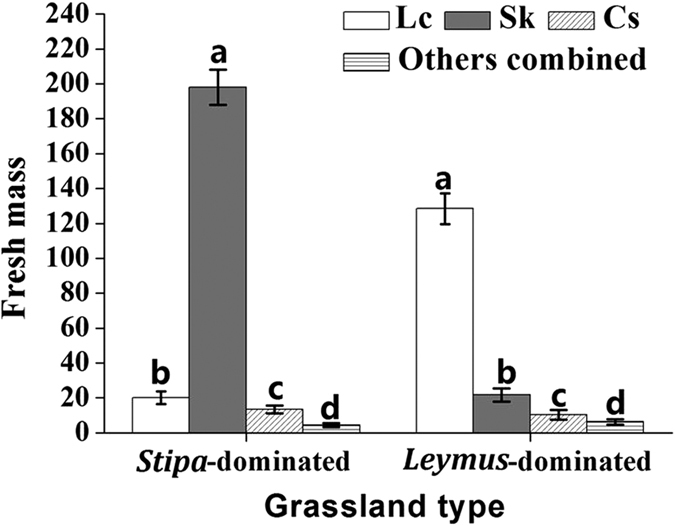
Composition (fresh mass per 1 m^2^, mean ± SD) of *L. chinensis* (Lc), *S. krylovii* (Sk), *C. squarrosa* (Cs) and other grass species in *Stipa*-dominated and *Leymus*-dominated grassland. ‘Other grass species’ comprised *A. frigida*, *A. cristatum*and *C. microphylla*, for *Stipa*-dominated grassland, and *A. frigida*, *C. microphylla*, *C. tragacanthoides* and *S. abrotanoides* in *Leymus*-dominated grassland. Bars marked by different lowercase letters are significantly different based on Turkey’s HSD analysis at *P* < 0.05.

Field surveys of our two grassland types showed that each contained three main grass species (*S. krylovii*, *L. chinensis*, and *C. squarrosa*), but at different biomass and composition ratios (Fig. 1). In the *Stipa*-dominated grassland, the fresh mass (n = 5; mean ± SD) and percent composition of the different grass species was in decreasing order *S. krylovii* (198.0 ± 10.1 g; ~83.4%) > *L. chinensis* (21.6 ± 3.6 g; ~9.2%) > *C. squarrosa* (13.4 ± 2.3 g; ~5.7%) > Other plant species (*Artemisia frigida* Willd., *Agropyron cristatum* (L.) Gaertn and *Caragana microphylla* Lam) (4.3 ± 1.1 g; ~1.7%). By comparison, in *Leymus*-dominated grassland, the fresh mass and percent composition of grasses was in decreasing order *L. chinensis* (128.4 ± 8.7 g; ~77.1%) > *S. krylovii* (21.6 ± 3.7 g; ~13.0%) > *C. squarrosa* (10.3 ± 2.9 g; ~6.1%) > other plant species (*A. frigida*, *Convolvulus tragacanthoides* Turcz., *C. microphylla*, and *Salsola abrotanoides* Bge.) (6.3 ± 1.5 g; ~3.8%).

**Figure 2:**
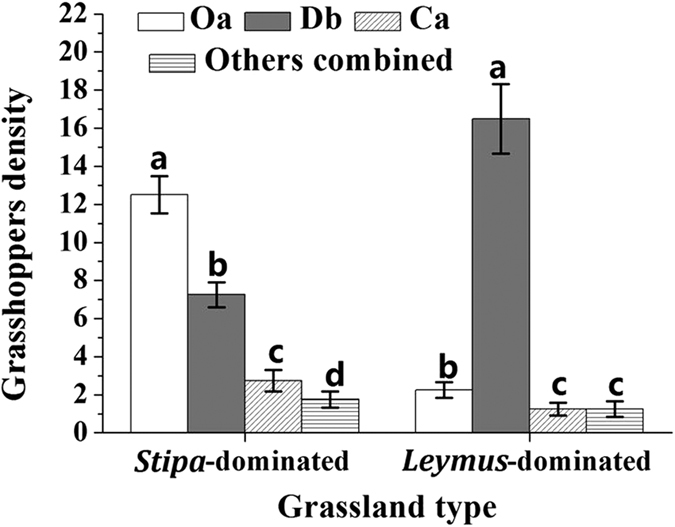
Grasshopper relative density (mean num ber of individuals (±SD) per 100 sweep-nets) in *Stipa*- and *Leymus*-dominated grassland, respectively. Oa = *O. asiaticus*, Db = *D. barbipes*, Ca = *C*. *abbreviatus*. In *Stipa-dominated* grassland ‘Other grasshopper species’ were *B. holdereri*, *B. luctuosum,* and *M. palpalis*. In *Leymus-dominated* grassland, ‘other grasshopper species’ were *B. luctuosum* and *M. palpalis*. Within each grassland, bars marked by different lowercase letters are significantly different based on Turkey’s HSD analysis at *P* < 0.05.

Similarly, while the two plots mainly contained three grasshopper species, *O. asiaticus*, *D. barbipes* and *C. abbreviates,* their relative density composition differed (Fig. 2). In *Stipa*-dominated grassland, a total of 96 grasshoppers were collected. The relative density (mean number of individuals ± SD/100 net-sweeps; n = 4) and percent composition of the species were *O. asiaticus* (12.5 ± 1.0; ~51.9%), *D. barbipes* (7.2 ± 0.7; ~29.8%), *C. abbreviatus* (2.7 ± 0.5; ~11.2%) with other grasshopper species consisting of *Bryodema holdereri holdereri* (Krauss), *Bryodema luctuosum* (Stoll) and *Myrmeleotettix palpalis* (Zub) (1.7 ± 0.4; ~7.1%). In *Leymus*-dominated grassland, a total of 85 grasshoppers were collected. The relative density and percent composition was in decreasing order *D. barbipes* (16.5 ± 1.8; ~78.2%), *O. asiaticus* (2.2 ± 0.4; ~10.4%), *C. abbreviatus* (1.2 ± 0.3; ~5.7%) and other grasshopper species (*B. luctuosum* and *M. palpalis*) (1.2 ± 0.4; ~5.7%). Thus, *O. asiaticus* was the primary grasshopper species in *Stipa*-dominated grassland, with *D. barbipes* the primary species in *Leymus*-dominated grassland.

### Standard curve

When plotting *C*_*t*_s against log_10_ of the grass isolate DNA copy number per reaction, the relationship was positive linear for all three grass species (*S. krylovii*: Y = 2.367x + 27.151, R^2^ = 0.984; *L. chinensis*: Y = 3.116x + 36.669, R^2^ = 0.999; *C. squarrosa*: Y = 3.111x + 37.003, R^2^ = 0.994). As calculated from the slopes of standard curves, the amplification efficiency (E) of *S. krylovii*, *L. chinensis* and *C. squarrosa* were 100.3%, 94.7%, 99.4%, respectively, all higher than 90%.

### Relationship between grass fresh mass and DNA copy number

There was a positive relationship for all three grass species between fresh mass against their mean log_10_DNA copy number across the 0.05–0.5 g weight range. For *S. krylovii*, the equation was: Y = 0.2275x − 3.986, R^2^ = 0.9206; for *L. chinensis*: Y = 0.0899x − 1.6275, R^2^ = 0.8775 and for *C. squarrosa*: Y = 0.0968x − 1.1829, R^2^ = 0.8486.

### Laboratory feeding trials

For *O. asiaticus* and *D. barbipes* grasshoppers, the detection efficiency for *L. chinensis*, *S. krylovii*, and *C. squarrosa* using real-time PCR following a single feeding period ranged from ~80 to ~97% (Table S1), indicating that this method was fairly accurate.

**Figure 3:**
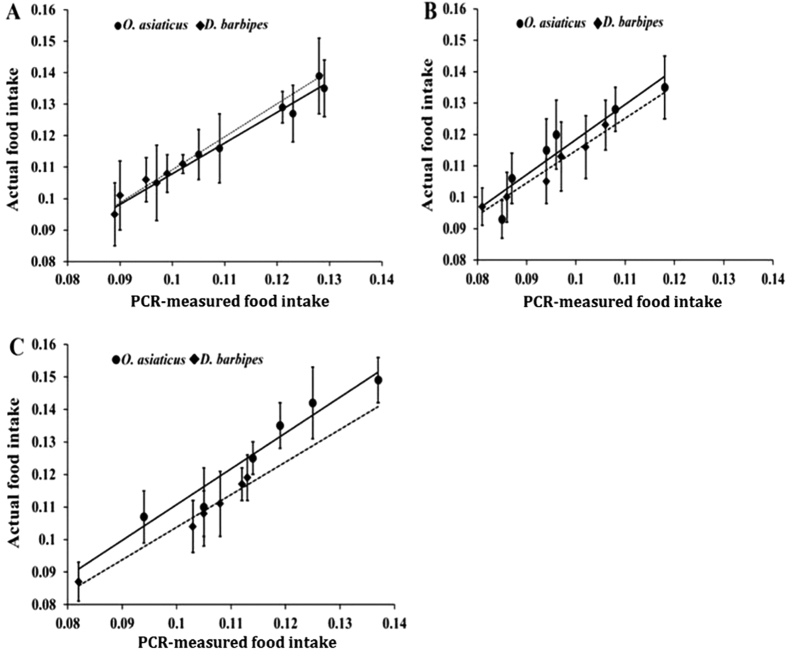
Relationship between actual food intake (g, mean ± SD) determined by weighing, and PCR-measured food intake (g, mean ± SD), determined by real-time PCR for *S.* *krylovii* (**A**), *L. chinensis* (**B)**,*C. squarrosa* (**C**) for the grasshopper species *O. asiaticus* (solid line) and *D. barbipes* (dashed line).

Linear-regression analysis comparing actual vs. PCR-estimated food intake by the two grasshopper species during the feeding period found a significant relationship for all three plant species. For the grass *S. krylovii* (Fig. 3A), the relationship for *O. asiaticus* was: y = 0.9748*x* + 0.0105, R^2^ = 0.942, *F* = 64.710, *P* = 0.001 and for *D. barbipes*: y = 1.0464*x* + 0.0046, R^2^ = 0.889, *F* = 31.964, *P* = 0.005. For the grass *L. chinensis* (Fig. 3B), the relationship for *O. asiaticus* was: y = 1.1173*x* + 0.0067, R^2^ = 0.879, *F* = 28.954, *P* = 0.001, and for *D. barbipes*: y = 1.0326*x* + 0.0116, R^2^ = 0.954, *F* = 83.860, *P* = 0.032. For the grass *C. squarrosa* (Fig. 3C), the relationship for *O. asiaticus* was described by the equation y = 1.006*x* + 0.0007, R^2^ = 0.945, *F* = 69.190, *P* = 0.001 and for *D. barbipes*: y = 1.0028*x* + 0.0035, R^2^ = 0.975, *F* = 1640625, *P* < 0.001. Hence, our real-time PCR method for quantifying grasshopper diets was accurate and reliable.

### Food intake by field collected grasshopper

**Figure 4:**
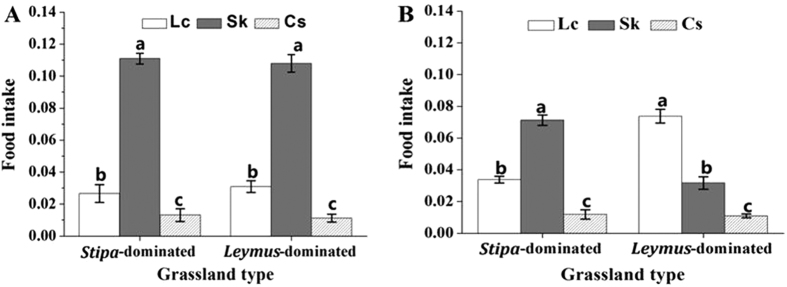
Food intake (g per female adult, means ± SD) of three grasses (Lc = *L. chinensis*, Sk = *S. krylovii*, and Cs = *C. squarrosa*) by *O. asiaticus* (**A)** and *D*. *barbipes* (**B**) in *Stipa*-dominated and *Leymus*-dominated grassland, respectively. Within each grassland, bars marked by different lowercase letters are significantly different based on Turkey’s HSD analysis at *P* < 0.05.

Using real-time PCR to quantify each grass fresh mass in the gut contents of grasshoppers collected from different grassland showed that *O. asiaticus* maintained a fixed diet structure (Fig. 4A) despite the ratio of plant species differing significantly between the two grassland habitats. In both *Leymus*- and *Stipa*- dominated grassland, the food intake by *O. asiaticus* was in decreasing order *S. krylovii* > *L. chinensis* > *C. squarrosa*, with an unchanged ~7:2:1 ratio (fixed diet structure) across the two grassland types. By comparison, *D. barbipes* changed its feeding pattern as plant species ratios changed. In *Stipa*-dominated grassland, *D. barbipes* fed mostly on *S. krylovii*. But, in *Leymus*-dominated grassland, *D. barbipes* fed mostly on *L. chinensis* (Fig 4B).

### Food preference analysis

**Figure 5:**
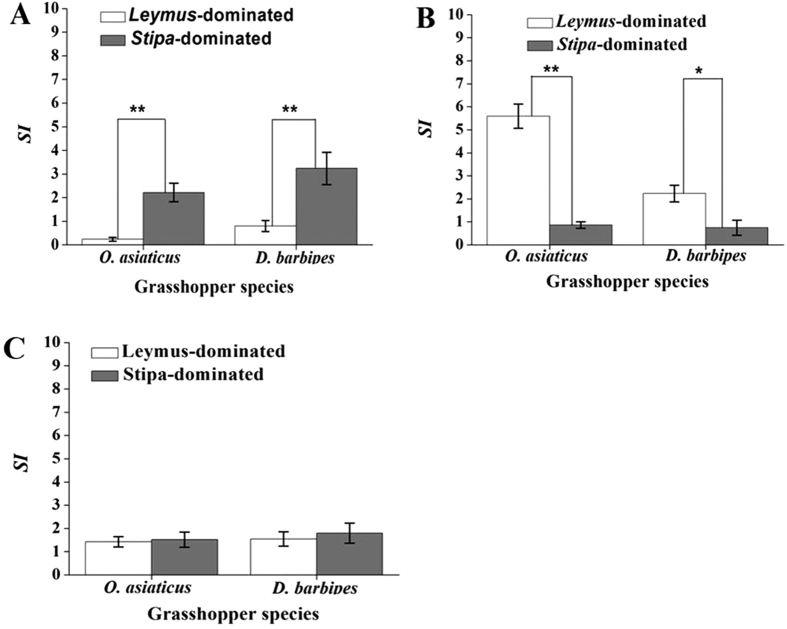
*O. asiaticus* and *D. barbipes* mean (±SD) diet selectivity index (*SI*) on *L. chinensis* (**A**) *S. krylovii* (**B**,**C**) *squarrosa* (**C**) in *Stipa*- and *Leymus*- dominated grassland. ‘*’ indicates that the values are significantly different at *P* < 0.05, ‘**’ indicates that the values significantly different at *P* < 0.01 (*t*-test).

Diet Selectivity Index (*SI)* analysis showed that the *SI* for *L. chinensis* in *Leymus*-dominant grassland with a high *L. chinensis* biomass was significantly less than in *Stipa*-dominant grasslands with little *L. chinensis (P* < 0.001) (Fig. 5A). This suggested that for both grasshopper species, food preference for *L. chinensis* increased in proportion to declining biomass. Similarly, the *SI* for *S. krylovii* in *Leymus*-dominated grassland with little *S. krylovii* biomass was significantly greater than in *Stipa*-dominated grassland with more *S. krylovii* biomass for both *O. asiaticus (P* < 0.001) and *D. barbipes (P* < 0.05) (Fig. 5B), which also suggested that grasshopper preference of *S. krylovii* increased with declining biomass. The *SI* for *C. squarrosa* (Fig. 5C), showed no significant difference for the approximate equal percent biomass in these two different plant communities.

## Discussion

Grasshoppers are important grassland and agricultural pests whose outbreaks can cause massive agricultural damage, leading to economic and social disruptions^2^,^7^,^44^. In order to control these pests, it is essential to be able to predict their spatial distribution and to understand the factors that lead to outbreaks. We know that diet influences grasshopper health, growth, development, longevity, reproduction, population dynamics, and spatial distributions^1^,^5^,^7^,^45^, and, as basic parameters of physiology, food intake and preference play important roles in constructing economic thresholds for these pests^46^. Understanding grasshopper diet structure and food preference allows us to predict their distributions and nutritional health, and thus improves our ability to control them. In this study, we demonstrate that real-time PCR on individual grasshoppers can illuminate food preference and feeding patterns. We also showed that our two grasshopper species differed in food preference and feeding patterns, which in turn offers some insight into their distributions.

The advantage of real-time PCR is that it provides the ability to evaluate food intake for individual grasshoppers where food intake is in comparatively small amounts^12^,^47^ as opposed to studies that investigate population-level impacts. Using designed steady ITS sequences targeting the three common host plants, we could ascertain both composition and quantity of food-plants in the grasshopper guts. In this study, we provided standard curves, which confirmed the linear relationships between C*t* values and each grass species DNA copy number, making the C*t* value a reliable way to quantify DNA copy number (in log10 form), and built the linear relationship between grass fresh mass and log_10_ of DNA copy number, making the log_10_ value a reliable way to quantify grasshopper food intake (measured food intake) of each grass. We also constructed the linear relationships between actual grasshopper food intake and PCR-measured food intake, making the measured food intake value a reliable method to quantify actual food intake during feeding period. Those provided the calibration equations and made the real-time PCR a reliable way for the diets structure studying of field samples.

In the laboratory feeding trial, although some of the PCR-measured food intake of the three host-plants were lower than actual food intake (Fig. 3), most of the food consumed within the 6-h feeding period was detected by real-time PCR for a relatively high detection efficiency (Table S1). The observed variance between actual food intake and PCR-measured food intake might be related to food digestion and DNA degradation during the 6-h feeding period^36^. Previous studies by Zhang *et al*.^39^,^40^ and Nejstgaard *et al*.^48^ also indicated that absolute estimates of gut content based on real-time PCR methodology were consistently lower than expected for many reasons, including the extraction methods used, potential interference by non-target DNA in the PCR reactions, the digestion of prey-specific nucleic acids of the time elapsed. Hence, we recommend that when using real-time PCR to analyze insect diets, short feeding periods be used, that gut samples be stabilized immediately after the feeding period, and that standard procedures be closely followed to prevent DNA degradation, and reduce error. Potential problems arising when using real-time PCR bioassays involving field-collected samples, include collection method, transport time to the laboratory, collection and transport conditions (e.g. temperature), grasshoppers size and DNA extraction technique, which can all influence digestion rates^40^,^48^,^49^,^50^,^51^. Hence, it is important to collect grasshoppers of similar sizes, maintain consistent conditions during transport for all samples, and to stabilize or analyze samples as quickly as possible. We also recommend using large sample sizes, when possible.

Our study suggests caution when using the PCR method to measure food intake over long feeding periods. This is because longer time intervals between ingestion and measurement may lead to greater variation and inaccuracies due to DNA degradation in the digestive system. In practice, we were able to collect individual grasshopper during feeding periods in the field, then, use the real-time PCR to rapidly determine the composition of their diet. While we only studied three food-plants of two grasshopper species, the success of the technique provides confidence to extend the technique to other grasshopper species and other host plants.

According to the calculated results of real-time PCR, the specific-fixed feeding pattern may explain why *O. asiaticus* is uncommon in *Leymus-*dominated habitats, but common at *Stipa-*dominated habitats (Fig. 2), which is consistent with the results of previous research by Cease *et al*.^2^. Thus, *O. asiaticus* control should be emphasized in *Stipa*-dominated grassland, and less so in *Leymus*-dominated habitats. By comparison, *D. barbipes* exhibited a more generalist feeding pattern in different grassland. The broader host range of *D. barbipes* perhaps indicates why this species is more widespread in Inner Mongolia^52^. In this study, the Diet Selectivity Index (*SI*) was also estimated using the quantification result of real time PCR. The *SI* value can more accurately reflect food preference of grasshoppers, because it includes information on both grasshopper consumption and plant community structure^53^ compared with traditional methods^12^,^22^,^54^. The results showed that when the mass of a specific food plant species mass declined, the grasshopper preference for this food plant (*SI*) increased. Previous research has already shown that when food resources become scarce, grasshopper populations often migrate^1^,^54^. As in *Leymus*-dominated grassland, grasshopper interspecific food competition for *S. krylovii* would become more acute as biomass declined compared with *Stipa*-dominated grassland where *S. krylovii* is more abundant. In our experiment, *O. asiaticus* retained a specific-fixed feeding pattern with a strong preference for *S. krylovii*, while the broader host range of *D. barbipes,* allows this grasshopper species to remain in *Leymus*-dominated grassland feeding on the other plant species present in the plant community. In *Stipa*-dominated grassland, interspecific food competition for *L. chinensis* would become more acute, commensurate with a significant decrease in the biomass of *L. chinensis*, which may result in the *D. barbipes* migration to *Leymus*-dominated grassland for general feeding pattern. Consequently, the relative density of *D. barbipes* in *Leymus*-dominated was significantly greater than in *Stipa*-dominated grassland (Fig. 2).

Studies of grasshoppers diet structure within plant communities are important for grasshopper control and protection of plant resources^48^,^55^,^56^. Using real-time PCR. we showed that (1) *O. asiaticus*, maintained a specific-fixed feeding pattern across two grassland habitats with differing grass communities, while the opposite was true for *D. barbipes*, which showed a generalist feeding pattern; (2) Preference for a particular host plant increased as overall biomass decreased, a response that not only maintains beneficial polyphagy, but would appear to increase interspecific competition which may drive migration. Such knowledge can aid rangeland and pest management. As such, overgrazing by sheep could decrease *L. chinensis* biomass, and increase the relative abundance of *S. krylovii*^57^,^58^. The change towards *Stipa*-dominated grassland, which may accelerate migration behaviour of *D. barbipes* to *Leymus*-dominant grassland, and consequently result in outbreak populations of *O. asiaticus* in *Stipa* dominant grasslands. These findings provided new insights that may improve livestock management strategies to limit the occurrence of economically damaging grasshopper outbreaks. Overall, our study confirms the potential of using real-time PCR to study diet structure and understand diet-induced grasshopper distribution, and therefore guide us for improved pest management.

## Materials & Methods

### Ethics Statement

No specific permissions were required at our sites, because they were not private and the study did not involve endangered or protected species.

### Study site

**Figure 6:**
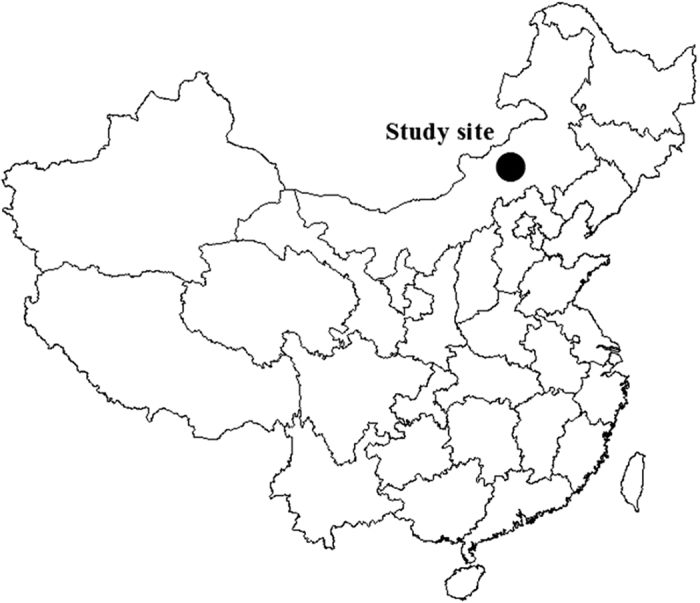
Map (created by ArcGIS version 10.2, http://www.esri.com/) indicating the location of field site is in the middle of Inner Mongolia (northern China).

The research site (43.968 N, 115.821 E) was located in the Xilin Gol League (Fig. 6), Inner Mongolia, northeast China, a region representative of the Eurasian steppe grassland and characterized by *Leymus*- and *Stipa*-dominated plant communities^2^. Rainfall from spring to fall is generally below 295 mm, and the annual average temperature 3.1 °C. Air temperatures can fall as low as −41 °C in December and reaches 35 °C in July. Rapid steppe degradation has led to reduced biodiversity, decreased productivity and, in some cases, desertification, attributed to livestock over-grazing^43^,^52^. Fifty-nine grasshopper species have been recorded from this area, but during June and July (summer time) three species: *Oedaleus asiaticus* B. Bienko, *Calliptamus abbreviatus* Ikonn. and *Dasyhippus barbipes* (Fischer-Waldheim), comprise ~ 80% of all grasshopper species^42^. These three species overwinter as eggs, and generally hatch between late-May and late-June, reaching adulthood between early to late July. From field observations, they fed mainly between 10:00–16:00 hrs (feeding period), when the sunshine is stronger and temperatures are warmer^47^.

### Plants and grasshopper composition of *Stipa*-dominated grassland and *Leymus*-dominated grassland in our study site

Two dominant grassland community types exist in our study area: *Leymus*-dominated and a *Stipa*-dominated. We characterized the plant and grasshopper composition of each by the following method: On July 10, 2013 (summer) we delineated a 1-km^2^ plot in each type of grassland. The two plots were ~5 km apart. In each plot, the plants within five 1-m^2^ quadrats (~50 m apart) were cut to ground level and each species in each quadrat placed separately into envelopes, then weighed. Fresh mass of different plant species of the five samples were averaged from each plot.

We also used sweep-net sampling to estimate the relative density of the two dominant grasshopper species in each of our two plots. For each plot, we started at the center, then walked nearly 500 m in each of the four cardinal directions, and conducted a standard sample of 100 sweeps with a 40-cm diameter sweep net. We recorded all grasshopper collected from each of the four directions, and averaged the four samples in each plot, to derive a relative grasshopper density (number of individuals per 100 sweep-nets) for each of the two plots.

### Plant material and DNA extraction

Foliage of *C. squarrosa, L. chinensis*, *S. krylovii*, *A. cristatum*, *C. microphylla*, *A. frigida*, *C. tragacanthoides* and *S. affinis* was collected from the field on 6 July 2013 and then stored within 24 hours in an ultra-cold storage freezer (−80 °C). In the laboratory, foliage from each plant species was placed in separate 1.5-mL micro centrifuge tubes and homogenized using a homogenizer in 200 μL of DNA extraction buffer (20 mM NaCl, 50 mM Tris–HCl, 1 mM EDTA, 1% SDS, and 20 mg mL^−1^ proteinase K, pH 8.0). The homogenate was agitated briefly and incubated in a water bath at 60 °C for 2 h, then boiled for 5 min to inactivate the proteinase K. After boiling, the lysate was extracted twice with one volume of 3.0 M potassium acetate, kept on ice for 1 h, and centrifuged for 15 min at 12,000 g at 4 °C. The aqueous phase was mixed with two volumes of ice-cold 100% ethanol. The precipitated DNA was centrifuged, dried, resuspended in 20 μl ultrapure water, and stored at −20 °C. DNA integrity was checked by agarose gel electrophoresis. DNA concentration and quality were assessed using a ND-1000 UV spectrophotometer (Nanodrop Technologies). All DNA extractions were performed in a room dedicated to the extraction of nucleic acids to avoid contamination, and conducted by one researcher alone.

### Primer design

‘Universal’ primers (Forward: 5′-CGTAACAAGGTTTCCGTAGG TGAAC-3′, Reverse: 5′-TTATTGATATGCTTAAACTCAGCGGG-3′)^59^ were used to amplify the ITS (internal transcribed spacer) of rDNA genes of three grass species. These primers were employed to characterize the 18S–28S rDNA region for the different grass species. Each PCR was carried out in 25 μL, containing 10 ng of template DNA; 0.25 μL Taq polymerase (5 U/μL, TaKaRa); 1 μL of each primer (Forward and Reverse); 4 μl dNTPs (2.5 mM); 2.5 μL 10 × Ex Taq Buffer (TaKaRa). Finally, we added ddH_2_O to bring the total volume to 25 μL. The PCRs were carried out in a GeneAmp 9700 thermocycler (Applied Biosystems). PCR cycling conditions used were 95 °C for 3 min followed by 35 cycles of 94 °C for 30 s, 55 °C for 30 s, 72 °C for 30 s, and a final cycle of 72 °C for 5 min. The PCR products were separated on a 2% low-melting-point agarose gel and electrophoresed (Bio-Rad, Sub-cell Model 192) for 0.5 h in 1 × TBE buffer at 120 v (Bio-Rad, Power Pac 1000). After sliced from the gel, the PCR product was purified (High Pure PCR Product Purification Kit; Shanghai Boya Biotechnological), ligated and cloned into pGEMT Easy Vector (Beijing Tianwei Shidai Biotechno-logical). The recombinant plasmids DNA were isolated from the obtained white colonies by using a High Pure Plasmid Isolation Kit (GenElute Plasmid Kits, Beijing Tianwei Shidai Biotechnological) according to the manufacturer’s instructions. The isolated recombinant plasmids were then sequenced (Yingjun Limited, Shanghai). According to the ITS region sequenced for each grass species, different loci were selected to design specific primers (Table 1) using Primer Express (Applied Biosystems). The primers were synthesized by Shanghai Gene Core Bio Technologies. The specificity of each primer pair was checked by singleplex real time PCR using iQ5 Sequence Detection System, respectively, with DNA from a broad range of other potential grass species in the study area, including *A. cristatum*, *C. microphylla*, *A. frigida*, *C. tragacanthoides* and *S. affinis*. In all cases, primers were found to be specific to the species for which they were designed (Fig. S1).

### Assay conditions of real-time PCR for quantification

We first optimized and identified the annealing temperatures of three grasses, using 60 °C for *S. krylovii*, 57 °C for *L. chinensis* and 55 °C for *C. squarrosa*. Then, singleplex PCR was optimized to amplify the three grass species ITS sequences, respectively. The PCR was carried out in iQ5 Sequence Detection System (Applied Biosystems, Bio-Rad company, American) using SYBR *Premix Ex Taq* II kit(TaKaRa). Following the manufacturer’s instructions, the real-time PCR protocol was performed in a final volume of 25 μL. Each tube contained: 2 μL sample DNA (10 ng); 12.5 μL SYBR *Premix Ex Taq* II; 1 μL (20 μM) of forward primer; 1 μL (20 μM) of reverse primer. Finally, we added ddH_2_O to provide a volume of 25 μL. The PCR protocol consisted of one step at 95 °C for 30 s, followed by 45 cycles at 94 °C for 20 s, the respective optimized annealing temperature (as described above) for 20 s, 72 °C for 20 s and a final cycle of 72 °C for 5 min. No-template controls were conducted to detect possible sample contamination. To ensure that only the desired product was amplified, a dissociation curve was produced for each reaction to monitor fluorescence continuously. All real-time PCR were performed in a room dedicated to the quantification to avoid contamination, and conducted by one researcher alone.

### Standard curve

The standard curve was generated using DNA from the purified plasmid isolated as the standard. Its concentration (μg/μL) was determined by spectrophotometric measurement (UV-visible Spectrophotometer, UV-2550, Shimadzu) and the numbers of target DNA sequence copies were calculated using the expression: the number of target DNA transcripts = [DNA mass (μg)/DNA molar mass] × 6.023 × 10^23^.

The number of transcripts was calculated for 10 ng, which was the volume used as the template in each real-time PCR assay. Through tenfold serial dilutions of the transcripts (dilution ratio 10^2^–10^8^), standard curve equation for the three grass species between *C*_*t*_ value and the copied amount of DNA were produced, respectively. Each point on the standard curve was assayed in triplicate. From the standard curve equation, we could determine the copy number of DNA (in log_10_ form) for each species according to the value of *C*_*t*_. Real time PCR amplification efficiencies (*E*) were calculated from slope values of standard curves using the formula −1 + 10^(−1 slop-1)^ (www.genequantification.de/mx4000-appnotes10.pdf).

### Relationship between grasses fresh mass and DNA copy number

For each species of grass, DNA was extracted from 0.05, 0.1, 0.2, 0.25, 0.3 and 0.5 g of plant material in 10 July 2013. The DNA copy number for each of the six weight samples was quantified with real-time PCR (as described above), with five replications per plant species. The mean copy number (in log_10_ form) of each grass species was calculated from their standard curve equations (as described above). The curve-fitting equation was derived from the DNA mean copy number (in log_10_ form) across the six sample weights. From these curve-fitting equation, we could conclude the fresh mass of each grass species according to the value of their DNA mean copy number (in log_10_ form).

### Laboratory feeding trials

Adult *O. asiaticus* were collected by sweep nets from the Xilingol rangeland at ~ 16:00 h on 10 July 2013, transferred to the laboratory, placed in aluminum metal cages (1 m × 1 m × 1 m) with a fine fabric mesh covering, and held without food overnight (18 h). Then, a subsample of ten starved individuals were collected before storing at −20 °C. In the morning, we collected fresh foliage of the three grass species (*S. krylovii*, *L. chinensis* and *C. squarrosa*). Fifty-four, 500-ml plastic vented containers were divided into three treatment groups according to the three grass species (18 containers per grass species). To study consumption, each container (replicate) received 0.5 g of fresh leaf of a single plant species, and two adult female *O. asiaticus*. The experiment ran for 6 h, from 10:00 to 16:00 hrs on 11 July 2003, in a controlled environment room at 30 °C under a 12:12 h light: dark regime, to mimic field conditions. After one hour (11:00 hrs), six live females from three containers were selected from each treatment, narcotized, and immediately dissected. Gut contents (eaten grass) were removed and placed in 100% ethanol before storing at −20 °C for real-time PCR quantification. At the same time, any uneaten grass still in the cages was collected, weighed, dried at 90 °C for 24 h, and then reweighed (*E*_*i*_). This was repeated at each hour for six hours until all containers were empty, at 16:00 hrs. Identical methods were used to study grass consumption by *D. barbipes*.

In parallel to the three feeding treatments, we also ran a control treatment, consisting of 54 cups without grasshoppers. One-half gram of plant material was placed in each control cup, and sampled hourly. Similar to treatments, we dried and weighed (*C*_*i*_) each grass species from each of the controls. This allowed us to calculate the baseline 1–6 h water loss for each of the three grass species, respectively. Consumption of fresh foliage (designated as actual food intake) for each grass species was calculated using the formula: 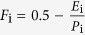, where *P*_i_ was the ratio of dry weight over wet weight 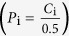 of the plant species, *E*_i_ was the dry weight of the plant species after feeding.

This allowed us to calculate the food intake of each grass species by each grasshopper species (designated as measured food intake). Finally, we compared and analyzed the relationship between the actual food intake and the PCR-measured food intake, and calculated the PCR detection efficiency (Detection Efficiency) during the feeding period by comparing PCR-measured food intake/actual food intake^39^,^40^.

The DNA of the starved and fed grasshoppers guts was extracted using the same method for plant DNA extraction described above. The starved grasshoppers were also verified using singleplex real time PCR to detect whether the three food plants could be amplified. Results showed that no band were found (Fig S1), suggesting that no food plants of the target species were amplified. Each gut sample was amplified individually with three technical replicates for quantification by real-time PCR.

### Food intake and preference by field collected grasshoppers

To determine the diet structure and food preference of the two grasshopper species in the field, sweep-netting was used to collect 18 female adults of each grasshoppers species from each grassland type (*Lemus*- and *Stipa*-dominated plant communities), on 15 July, 2013 at 15:00 hrs. Grasshoppers were transferred to containers and returned to our field laboratory (Scientific Observation and Experimental Station of Pests in Xilin Gol Rangeland) immediately, this process took ~ 10 min. There they were narcotized and their gut contents were removed and placed immediately in 100% ethanol before storing at −20 °C for real-time PCR quantification. The food intake (measured value) of the three grass species was calculated using the curve-fitting equation for the fresh mass of each species and their respective DNA copy numbers (as described above). Actual food intake was then derived from the measured food intake as determined by real-time PCR. Total 36 grasshoppers in each grassland type were analyzed.

### Data analysis

We analyzed the relationships between actual and PCR-measured food intake derived from the laboratory feeding trails experiments. We used Student’s *t-*test to compare grasshopper density in *Stipa*- and *Leymus*-dominated grassland. One-way analysis of variance (ANOVA) was used to compare the food intake of three grasses species by *O. asiaticus* and *D. barbipes* in the field samples experiment, respectively. We used SAS version 8.0 for all analyses.

The Diet Selectivity Index (*SI*) for field collected *O. asiaticus* and *D. barbipes* was determined according to the formula^53^,^58^.


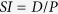
, where *D* = percentage of a plant species consumed and *P* = percentage of biomass of the same species in the environment. A *SI* of 1 indicates that the plant species was consumed in the same ratio as its availability (i.e., no preference). A *SI* > 1 indicates that a greater proportion of that plant species was eaten than was available, and thus was a preferred food for the herbivore. A *SI* < 1 indicates that the herbivore ate a smaller proportion of that species, than was presented, and thus that plant was less preferred by the herbivore.

## Additional Information

**How to cite this article**: Huang, X. *et al*. Quantitative analysis of diet structure by real-time PCR, reveals different feeding patterns by two dominant grasshopper species. *Sci. Rep.* **6**, 32166; doi: 10.1038/srep32166 (2016).

## Supplementary Material

Supplementary Information

## Figures and Tables

**Table 1:** Designed sequences of real-time PCR primers (ITS sequence) for the three grass species eaten by the grasshoppers *O.asiaticus* and *D. barbipes*.

**Grass species**	**Sequence of primers for real-time PCR (5′ to 3′)**		**Length (bp)**
*S. krylovii*	Forward	GGCACAGCGCGTGGTGGATCTC	145
	Reverse	TGCTTAAACTCAGCGGGTAGTC	
*L. chinensis*	Forward	AATCGGGATGCGGCATC	134
	Reverse	CATCCGACGCGTAGCCG	
*C. squarrosa*	Forward	CTGTGCAGCGATGCTATGA	229
	Reverse	TGCGGACGTGGTGTTTG	
