# Supplementary material for: Quantitative analysis of diet structure by real-time PCR, reveals different feeding patterns by two dominant grasshopper species
Source: Sci Rep. 2016 Aug 26;6:32166. doi: 10.1038/srep32166 (PMC4999873; doi:10.1038/srep32166)
Supplement: Supplementary Information [file srep32166-s1.pdf]

Quantitative analysis of diet structure by real-time PCR, reveals different feeding patterns by two dominant grasshopper species

Xunbing Huang[1, 2], Huihui Wu[1,2] , Mark Richard McNeill[3], Xinghu Qin[1,2], Jingchuan Ma[1,2], Xiongbing Tu[1,2], Guangchun Cao[1,2], Guangjun Wang[1,2], Xiangqun Nong[1,2] & Zehua Zhang[1, 2]*

Table S1 The measured food intake by PCR (g±SD), actual food intake by weighing method (g±SD) and detection efficiency (%) of *Stipa krylovii*, *Leymus chinensis*, *Cleistogenes squarrosa* by *O. asiaticus* (Oa)and *D. barbipes* (Db) during feeding periods (1000 hrs - 1600 hrs) in laboratory feeding trials

Species	Feeding hours (h)	*S. krylovii*			*L. chinensis*			*C. squarrosa*		
		PCR (g)	Actual (g)	Detection efficience	PCR (g)	Actual (g)	Detection efficience	PCR (g)	Actual (g)	Detection efficience
Oa	1	0.105± 0.004	0.114± 0.008	92.11	0.085 ± 0.008	0.093± 0.006	91.40	0.094 ± 0.004	0.107 ± 0.008	87.85
	2	0.109± 0.007	0.116± 0.011	93.97	0.087 ± 0.007	0.106± 0.008	82.08	0.105 ± 0.004	0.110 ± 0.012	95.45
	3	0.123± 0.004	0.127± 0.009	96.85	0.094 ± 0.005	0.115± 0.010	81.74	0.114 ± 0.008	0.125 ± 0.005	91.20
	4	0.129± 0.008	0.135± 0.009	95.56	0.096 ± 0.011	0.12± 0.011	80.00	0.119 ± 0.005	0.135 ± 0.007	88.15
	5	0.128± 0.012	0.139± 0.012	92.09	0.108 ± 0.009	0.128± 0.007	84.38	0.125 ± 0.010	0.142 ± 0.011	88.03
	6	0.121± 0.011	0.129± 0.005	93.80	0.118 ± 0.009	0.135± 0.010	87.41	0.137 ± 0.006	0.149 ± 0.007	91.95
Db	1	0.089± 0.011	0.095± 0.010	93.68	0.081 ± 0.006	0.097± 0.006	83.51	0.082 ± 0.005	0.087 ± 0.006	94.25
	2	0.090± 0.009	0.101± 0.011	89.11	0.086 ± 0.005	0.100± 0.008	86.00	0.103 ± 0.005	0.104 ± 0.008	99.04
	3	0.095± 0.005	0.106± 0.007	89.62	0.094 ± 0.009	0.105± 0.007	89.52	0.105 ± 0.006	0.108 ± 0.007	97.22
	4	0.097± 0.007	0.105± 0.012	92.38	0.097 ± 0.007	0.113± 0.011	85.84	0.108 ± 0.003	0.111 ± 0.010	97.30
	5	0.099± 0.006	0.108± 0.006	91.67	0.102 ± 0.008	0.116± 0.010	87.93	0.112 ± 0.009	0.117 ± 0.005	95.73
	6	0.102± 0.004	0.111± 0.003	91.89	0.106 ± 0.007	0.123± 0.008	86.18	0.113 ± 0.011	0.119 ± 0.007	94.96

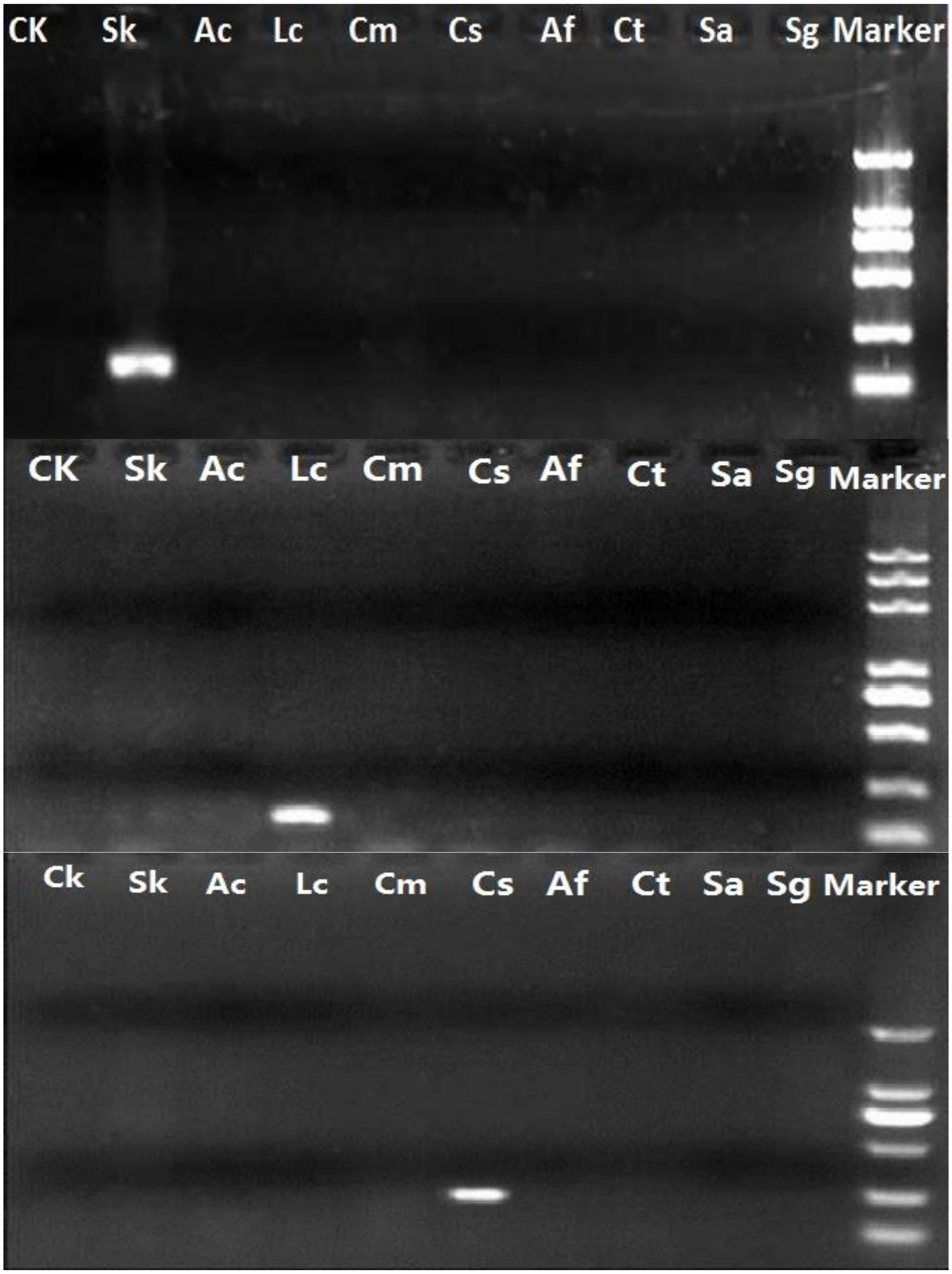

Fig. S1 The specificity of each grass primers by singleplex real time PCR using iQ5. The abbreviations Sk, Ac, Lc, Cm, Cs, Af, Ct, Sa, Sg represent plant species *Stipa krylovii*, *Agropyron cristatum*, *Leymus chinensis*, *Caragana microphylla*, *Cleistogenes squarrosa*, *Artemisia frigida*, *Convolvulus tragacanthoides*, *Salsola affinis*, and the starved grasshoppers, respectively.
